# A focal extramedullary hematopoiesis of the spleen in a patient with essential thrombocythemia presenting with a complicated postoperative course: a case report

**DOI:** 10.1186/s40792-021-01119-5

**Published:** 2021-01-26

**Authors:** Kiyotaka Hosoda, Akira Shimizu, Koji Kubota, Tsuyoshi Notake, Shinsuke Sugenoya, Koya Yasukawa, Hikaru Hayashi, Ryoichiro Kobayashi, Yuji Soejima

**Affiliations:** Division of Gastroenterological, Hepato-Biliary-Pancreatic, Transplantation and Pediatric Surgery, Department of Surgery, Shinshu University School of Medicine, 3-1-1 Asahi, Matsumoto, Nagano 390-8621 Japan

**Keywords:** Extramedullary hematopoiesis, Essential thrombocythemia, Splenectomy

## Abstract

**Background:**

Extramedullary hematopoiesis is a compensatory response occurring secondary to inadequate bone marrow function and is occasionally observed in essential thrombocythemia (ET). This disease usually presents as multifocal masses in the paravertebral or intra-abdominal region; however, formation of a focal mass in the liver or spleen is rare. In addition, ET is characterized by increased platelet count and shows a tendency toward thrombosis and, occasionally, bleeding. Serious bleeding is common in ET patients, caused by the decrease in or abnormalities of von Willebrand factor (vWF) as a consequence of the precipitous rise in platelets. Therefore, strict management of platelet count using medication is crucial in patients with ET who require invasive procedures, especially splenectomy.

**Case presentation:**

A 68-year-old man with ET was found to have an enlargement of a focal splenic tumor. Imaging findings revealed that the tumor was likely a hemangioma or hamartoma; however, the possibility of malignant disease could not be completely ruled out because of short-term tumor enlargement, and we conducted a splenectomy. The surgery was uneventful, but the patient presented with severe polycythemia and vWF abnormalities postoperatively, which resulted in bleeding from the drain insertion site and wound, epistaxis, and hemorrhoidal bleeding. Three months after discharge, polycythemia still persisted and the level of vWF gradually decreased. With a decrease in vWF, the patient suffered from an increased bleeding tendency. Therefore, the patient has been referred for bone marrow transplantation and is currently awaiting a suitable donor.

**Conclusions:**

Extramedullary hematopoiesis should be listed as a differential diagnosis of focal enlarged splenic tumors, especially in patients with myeloproliferative disorders. Additionally, in splenectomy for ET patients, careful perioperative management taking into consideration the conflicting features of a tendency toward thrombus formation and bleeding is necessary.

## Background

Extramedullary hematopoiesis (EMH) is a compensatory response occurring secondary to inadequate bone marrow function. EMH is observed in hematological disorders such as congenital hemolytic anemia, thalassemia, myelofibrosis, or leukemia, and occasionally in essential thrombocythemia (ET). This disease usually presents as multifocal masses in the paravertebral region or intra-abdominal region; however, formation of a focal mass in the liver or spleen is rare, and only a few cases have been previously reported [1].

As for ET, this disease is characterized by increased platelet count and a tendency toward thrombosis and occasional bleeding. Serious bleeding tendency is often seen in ET patients, caused by the decrease in or abnormalities of von Willebrand factor (vWF) as a consequence of the precipitous rise in platelets [2]. Therefore, strict management of platelet count is necessary in cases requiring invasive procedures. We report a case in which medication could not be administered because of an adverse event or drug resistance, resulting in severe polycythemia and bleeding tendency after splenectomy. Herein, the clinical features, including image findings and intriguing postoperative course, are presented.

## Case presentation

A 68-year-old man with ET was found to have an enlargement of a splenic tumor detected by enhanced computed tomography (CT) scanning, and was admitted to our institution for further investigation. The patient had a history of ET, hypertension, and acute heart failure induced by anagrelide, which is used to treat ET. He was treated with aspirin for ET because he acquired drug resistance to hydroxycarbamide, which is widely used for cytoreductive therapy in ET patients along with anagrelide. We diagnosed that bone marrow transplantation was required in the foreseeable future. He also regularly underwent imaging tests for renal tumors suspected to have complicated cysts.

Upon admission, he had no bleeding tendency. The complete blood count revealed leukocytosis and thrombocytosis (white blood cell (WBC) count: 23.4 × 10^3^ µL; platelet count: 854 × 10^3^ µL). Anemia and erythrocytosis (hemoglobin: 15.9 g/dL) were not detected. Although serum lactate dehydrogenase levels and serum soluble interleukin 2 receptor levels were elevated (1431 IU/ml and 887 IU/ml, respectively), other laboratory data were almost within normal limits.

**Fig. 1:**
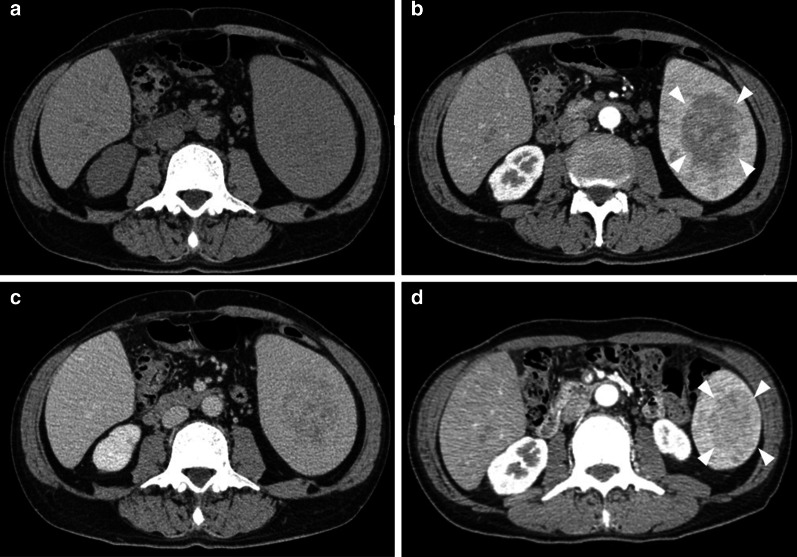
Computed tomography scan of the tumor. A computed tomography scan on admission showed a 70 × 45 mm low-density tumor (**a**). The tumor exhibited heterogeneous hypo-enhancement during the arterial phase (**b** arrowheads), and thereafter, the enhancement became gradually homogeneous in the equilibrium phase (**c**). The tumor was 45 × 30 mm in diameter on a preoperative CT scan 6 months before the surgery (**d** arrowheads)

**Fig. 2:**
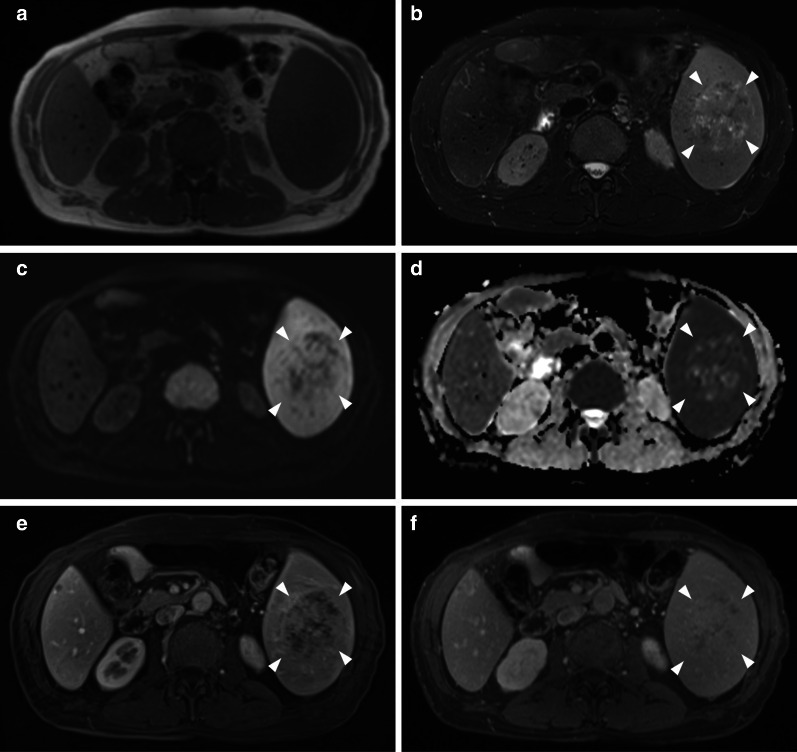
Magnetic resonance imaging of the tumor. Magnetic resonance imaging showed that the tumor signal was iso-intense on T1-weighted imaging **a** and exhibited a mottled high-intensity on T2-weighted imaging (**b** arrowheads), low-intensity on diffusion-weighted imaging (**c** arrowheads), and high-intensity on apparent diffusion coefficient mapping (**d** arrowheads). Dynamic studies revealed an enhancement pattern comparable to computed tomography scan (**e**, **f** arrowheads)

A CT scan showed a 70 × 45 mm low-density tumor in the spleen. The tumor exhibited heterogeneous hypo-enhancement during the arterial phase, and thereafter, the enhancement became gradually homogeneous. The tumor had grown from 45 to 70 mm in diameter in 6 months (Fig. 1). Magnetic resonance imaging (MRI) showed that the tumor signal was iso-intense on T1-weighted imaging (T1WI), exhibited a mottled high-intensity on T2-weighted imaging (T2WI), and exhibited low-intensity on diffusion-weighted imaging (Fig. 2). Fluorodeoxyglucose (FDG)-positron emission tomography/CT showed moderate FDG uptake with a maximum standardized uptake value of 4.9.

Based on these findings, hemangioma or hamartoma were the most likely options for differential diagnosis; however, the possibility of malignant disease could not be completely ruled out because of short-term enlargement of the tumor. Thus, we performed a splenectomy.

**Fig. 3:**
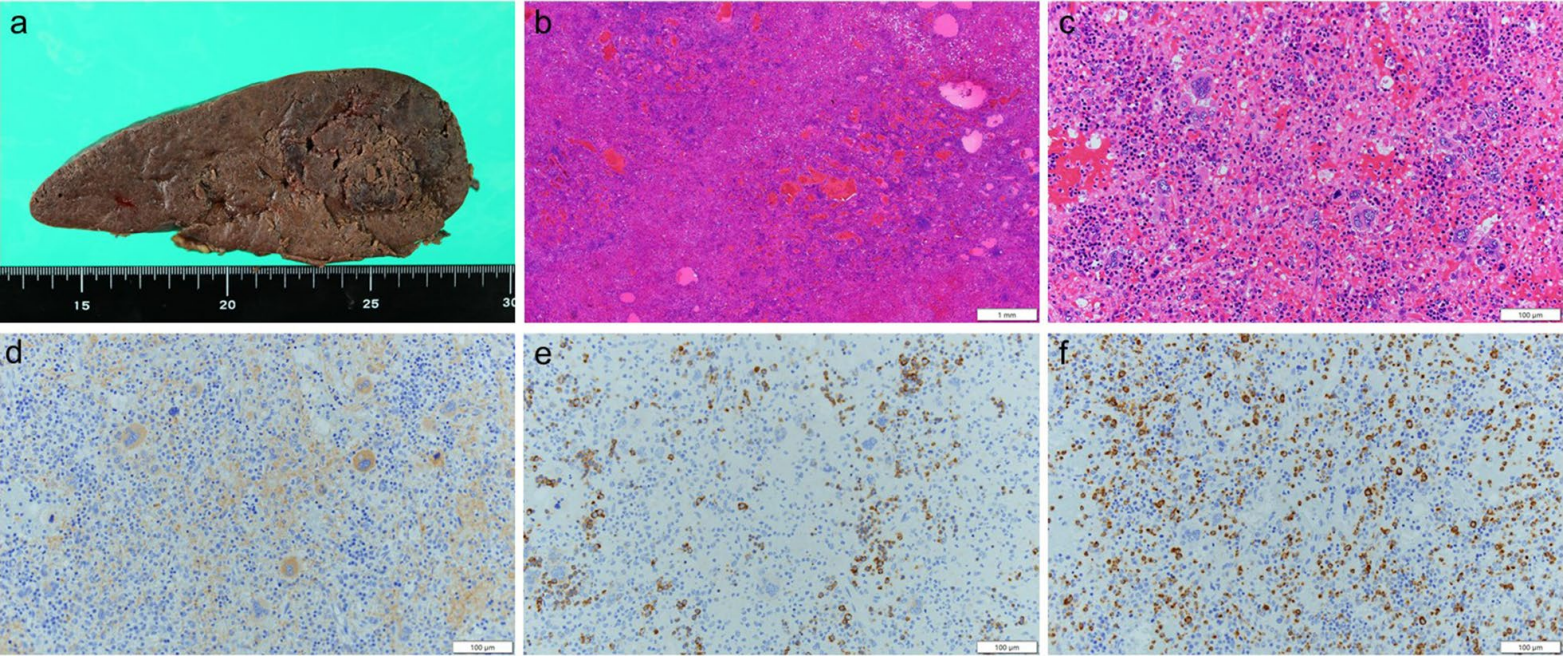
Histologic examination and immunohistochemistry of the tumor. Histologic examination revealed a 125 × 45 mm poorly marginated tumor (**a** arrowheads). Outgrowths of granular cells and erythroid cells were observed in the tumor [hematoxylin and eosin stain, × 2 (**b**), × 20 (**c**)]. Immunohistochemistry was positive for CD 71 (**d**), CD 41 (**e**), myeloperoxidase (**f**), and hematopoietic cells, including erythroblasts, granulocytes, and megakaryocytes (× 20)

Intraoperative findings showed marked splenomegaly. Liver cirrhosis, liver tumor, or lymphadenopathy were not detected. Histologic examination revealed a 125 × 45 mm poorly marginated tumor (Fig. 3a), and outgrowths of granular cells and erythroid cells were observed in the tumor (Fig. 3b, c). Immunohistochemistry was positive for CD 71, CD 41, and myeloperoxidase (Fig. 3d, e), and hematopoietic cells, including erythroblasts, granulocytes, and megakaryocytes, were observed. Based on these findings, focal extramedullary hematopoiesis of the spleen was diagnosed.

**Fig. 4:**
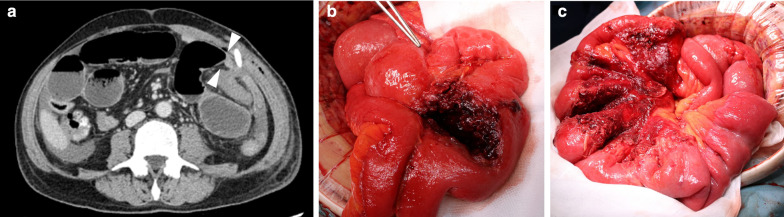
Computed tomography scan before re-operation and operative finding. Computed tomography revealed intra-abdominal hematoma and caliber changes in the small intestine (**a** arrowheads). The small intestine was obstructed by the intra-abdominal hematoma (**b**), and intestinal obstruction was released by removal of the hematoma (**c**)

The surgery was uneventful, and the patient began prophylactic administration of heparin immediately after surgery to prevent thrombosis. However, he presented with severe polycythemia (platelet count: 2600 × 10^3^ µL on postoperative day (POD) 8) and abnormalities of vWF (vWF activity: 46% on POD 17) after surgery, which resulted in bleeding tendency (bleeding from drain insertion site on the day of surgery, epistaxis on POD 14, and hemorrhoidal bleeding on POD 15) despite discontinuation of heparin administration. We did not provide vWF supplementation since administration of vWF in patients with thrombocytosis can promote severe platelet aggregation. Bleeding from the drain insertion site resulted in intra-abdominal hematoma and intestinal obstruction (Fig. 4); the obstruction was released on POD 10.

**Fig. 5:**
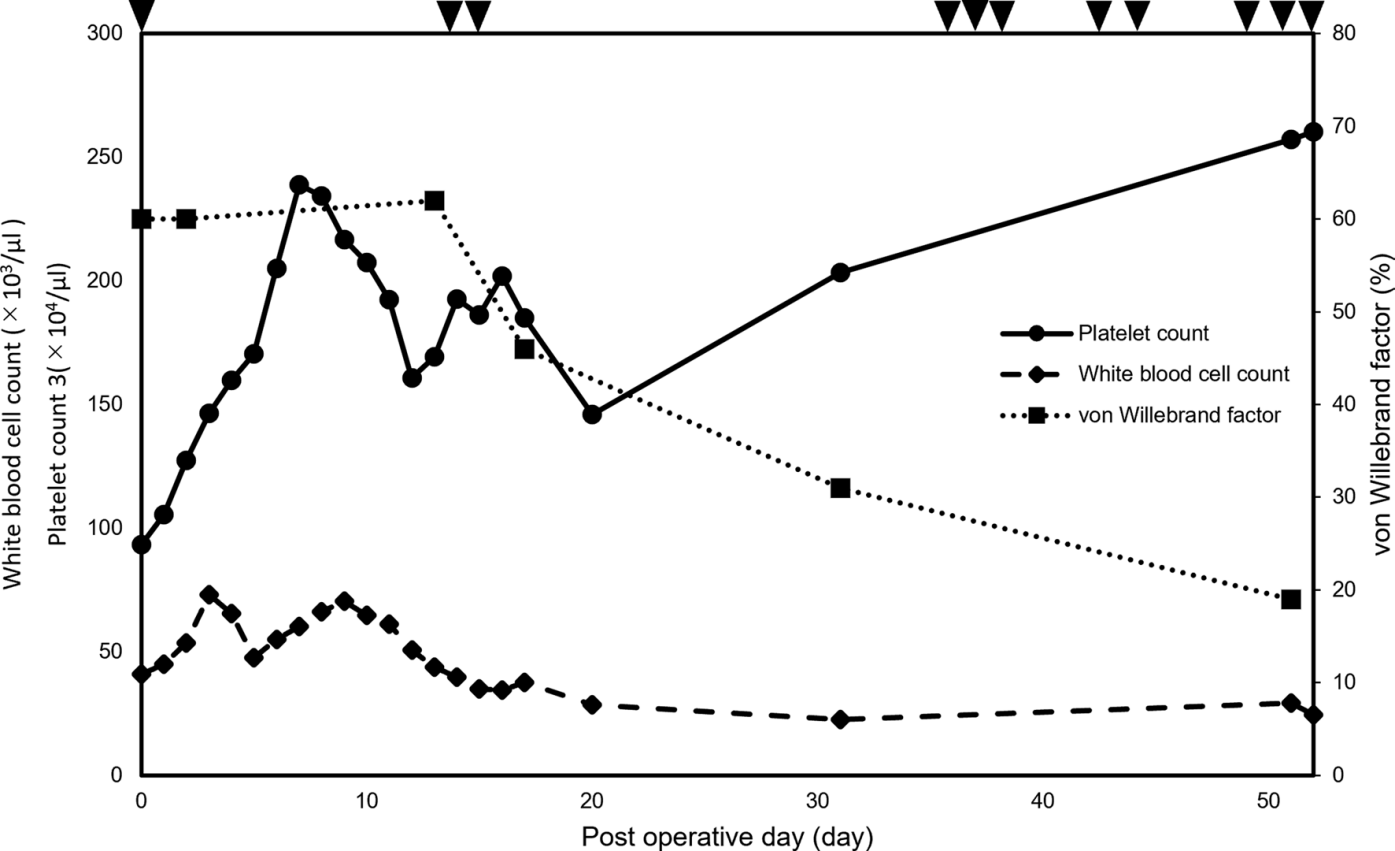
Postoperative white blood cell count, platelet count, and von Willebrand factor level. Postoperatively, platelet count gradually increased and von Willebrand factor decreased. With a decrease in von Willebrand factor, the patient repeatedly suffered from bleeding events (arrowhead). White blood cell count increased in the perioperative period and gradually decreased thereafter

Following re-operation and hemostasis of epistaxis and hemorrhoidal bleeding, the patient recovered smoothly and was discharged on POD 22. At the time of writing, 3 months have passed since discharge, yet polycythemia persists and the level of vWF is gradually decreasing (platelet count and vWF activity: 2030 × 10^3^ µL and 31% on POD 31 and 2571 × 10^3^ µL and 19% on POD 51, respectively). We tried to administer hydroxycarbamide, with which the patient previously showed drug resistance, but thrombocytosis did not improve. With a decrease in vWF, the patient repeatedly suffered from epistaxis and bleeding from the surgical wound or slight abrasion (Fig. 5). The patient is currently awaiting bone marrow transplantation.

## Discussion

EMH is a compensatory response that occurs secondary to inadequate bone marrow function. EMH is usually found as a multifocal mass in the paravertebral region or intra-abdominal region, and although quite rare, EMH has been reported to present as a focal mass in the spleen [1, 3–8], which could induce short-term enlargement. Regarding the image findings, it has been reported that EMH presents as homogenous or slightly heterogeneous low-density lesions with little or no enhancement on CT [3, 5–7], which are visualized with low to iso-intensity on T1WI and low to high intensity on T2WI [4, 7]. As these findings are nonspecific, EMH is difficult to distinguish from focal splenic tumors such as hemangioma, hamartoma, littoral cell angioma, lymphangioma, inflammatory pseudotumor, malignant lymphoma, metastases, or hemangiosarcoma [9] by imaging findings alone. This is because the image patterns of focal splenic tumors overlap between these tumors, and CT and MRI findings have variable characteristics depending on the status of EMH [4, 7, 8]. However, the combination of these imaging findings and medical history, including history of hematological disorders, hemolytic anemia, thalassemia, myelofibrosis, leukemia, or ET, could suggest the possibility of EMH. In diagnosing splenic EMH, imaging findings are insufficient for definitive diagnosis, and histological examination is necessary. As biopsy for splenic EMH is a risky procedure, splenectomy is recommended. In ET patients, however, total splenectomy might cause various complications, as in this case, and partial splenectomy and biopsy, by laparotomy or laparoscopy in some cases, may be considered as a treatment option, especially in cases where primary splenic malignancy is not strongly suspected. It is because partial splenectomy can suppress the increase in platelet and decrease in vWF levels by preserving functions of the reticuloendothelial system, and bleeding tendency can be prevented.

We now consider the clinical course of this patient, which suggests the following important clinical issues. In general, ET patients who undergo splenectomy have a higher risk of polycythemia and a severe bleeding tendency [10]. ET is a myeloproliferative disorder characterized by increased platelet count and a tendency to thrombosis and occasional bleeding. It is noted that the bleeding tendency of this disease is associated with a decrease in or disorder of vWF, especially the macromolecule multimer of vWF. vWF exists as multimers of various sizes and is adsorbed on the surface of platelets. Macromolecule multimers of vWF have a better hemostatic ability than that of other sizes, and when a large number of these molecules are absorbed or become deformed due to an increase in platelet count, a serious bleeding tendency can develop [2, 11, 12]. Hence, careful attention to bleeding tendency in patients requiring invasive procedures is important, especially in those undergoing splenectomy with a marked increase in platelet levels and splenomegaly due to ET, and strict management of platelet count, using medication such as anagrelide or hydroxycarbamide, is necessary [10]. However, patients who cannot receive these medications preoperatively, because of medication allergy, adverse events, or drug resistance, have a high possibility of polycythemia, abnormalities of vWF, and severe bleeding tendency. In such cases, the possibility of bone marrow transplantation should be constantly considered. The short-term use of busulfan can be a therapeutic option as preoperative management; however, chronic administration should be avoided because of secondary carcinogenesis. Thrombocytapheresis can also be an option for emergency situations [13]. Our patient could not receive both anagrelide and busulfan because of adverse events and deterioration of chronic renal failure, respectively, and the patient exhibited drug resistance to hydroxycarbamide. There was loss of phagocytic capacity after splenectomy, the increase in platelets became uncontrollable even in the late phase, and the platelet count gradually increased to 2600 × 10^3^ µL while the vWF decreased. The patient exhibited a significant bleeding tendency and now requires a bone marrow transplant. To prevent cases like this in the future, it is necessary to list EMH as a differential diagnosis of a focal splenic mass and to carefully consider the selection of treatment, including partial splenectomy or biopsy.

To our knowledge, no previous reports have detailed the clinical course of splenectomy in an ET patient from the immediate postoperative period to a few months postoperatively.

## Conclusions

EMH can present as a focal mass in the spleen, and EMH should be listed as one of the differential diagnoses of focal enlarged splenic tumors, especially in patients with myeloproliferative disorders. Furthermore, in splenectomy for ET patients, careful perioperative management taking into consideration the conflicting features of a tendency toward thrombus formation and bleeding in necessary.

## Data Availability

All data generated during this study are included in this published article.

## Abbreviations

CT: Computed tomography
EMH: Extramedullary hematopoiesis
ET: Essential thrombocythemia
FDG: Fluorodeoxyglucose
MRI: Magnetic resonance imaging
POD: Postoperative day
T1WI: T1-weighted imaging
T2WI: T2-weighted imaging
vWF: von Willebrand factor
WBC: White blood cell
